# Skeletal Muscle Protein Composition Adaptations to 10 Weeks of High-Load Resistance Training in Previously-Trained Males

**DOI:** 10.3389/fphys.2020.00259

**Published:** 2020-03-26

**Authors:** Christopher G. Vann, Shelby C. Osburn, Petey W. Mumford, Paul A. Roberson, Carlton D. Fox, Casey L. Sexton, McLelland-Rae Johnson, Joel S. Johnson, Jacob Shake, Johnathon H. Moore, Kevin Millevoi, Darren T. Beck, Veera L. D. Badisa, Benjamin M. Mwashote, Victor Ibeanusi, Rakesh K. Singh, Michael D. Roberts

**Affiliations:** ^1^School of Kinesiology, Auburn University, Auburn, AL, United States; ^2^Department of Exercise Science, LaGrange College, LaGrange, GA, United States; ^3^School of the Environment, Florida A&M University, Tallahassee, FL, United States; ^4^Edward Via College of Osteopathic Medicine Auburn, Auburn, AL, United States; ^5^Translational Science Laboratory, College of Medicine, Florida State University, Tallahassee, FL, United States

**Keywords:** high-load training, actin, myosin, proteomics, skeletal muscle

## Abstract

While high-load resistance training increases muscle hypertrophy, the intramuscular protein responses to this form of training remains largely unknown. In the current study, recreationally resistance-trained college-aged males (*N* = 15; mean ± SD: 23 ± 3 years old, 6 ± 5 years training) performed full-body, low-volume, high-load [68–90% of one repetition maximum (1RM)] resistance training over 10 weeks. Back squat strength testing, body composition testing, and a vastus lateralis biopsy were performed before (PRE) and 72 h after the 10-week training program (POST). Fiber type-specific cross-sectional area (fCSA), myofibrillar protein concentrations, sarcoplasmic protein concentrations, myosin heavy chain and actin protein abundances, and muscle tissue percent fluid were analyzed. The abundances of individual sarcoplasmic proteins in 10 of the 15 participants were also assessed using proteomics. Significant increases (*p* < 0.05) in type II fCSA and back squat strength occurred with training, although whole-body fat-free mass paradoxically decreased (*p* = 0.026). No changes in sarcoplasmic protein concentrations or muscle tissue percent fluid were observed. Myosin heavy chain protein abundance trended downward (−2.9 ± 5.8%, *p* = 0.069) and actin protein abundance decreased (−3.2 ± 5.3%, *p* = 0.034) with training. Proteomics indicated only 13 sarcoplasmic proteins were altered with training (12 up-regulated, 1 down-regulated, *p* < 0.05). Bioinformatics indicated no signaling pathways were affected, and proteins involved with metabolism (e.g., ATP-PCr, glycolysis, TCA cycle, or beta-oxidation) were not affected. These data comprehensively describe intramuscular protein adaptations that occur following 10 weeks of high-load resistance training. Although previous data from our laboratory suggests high-volume resistance training enhances the ATP-PCr and glycolytic pathways, we observed different changes in metabolism-related proteins in the current study with high-load training.

## Introduction

High-load resistance exercise involves performing lifts with heavier weights for fewer repetitions [sets consisting of 1–6 repetitions at ≥75% one repetition maximum (1RM)]. Moderate-to-higher volume resistance exercise involves performing more repetitions using light to moderate loads (sets consisting of >10 repetitions at ≤65% 1RM). There has been recent enthusiasm surrounding molecular signaling events that occur in skeletal muscle in response to higher load versus moderate-to-higher volume resistance exercise (Burd et al., 2010; Haun et al., 2017; Morton et al., 2019). In general, these studies have reported that acute anabolic signaling events do not differ between exercise modalities when lifts are performed to volitional fatigue. In addition, a review that analyzed 11 studies reported similar increases in mean fiber cross-sectional area (fCSA) and types I and II fCSA in response to months of higher versus lower-load resistance training (Grgic and Schoenfeld, 2018). However, the intramuscular protein adaptations that occur in response to each mode of training – specifically, changes in myofibrillar protein concentrations, sarcoplasmic protein concentrations, or muscle tissue percent fluid – have been vastly understudied.

Indeed, there have been studies that have examined how resistance training affects certain protein adaptations (e.g., myofibrillar protein concentrations, sarcoplasmic protein concentrations, or muscle tissue percent fluid) along with hypertrophy measures (e.g., changes in fCSA or localized measurements of muscle hypertrophy) [reviewed in Haun et al. (2019b)]. While insightful, there were limitations including: (a) some studies contained fewer than eight participants, (b) some studies included participants with diseases, (c) some studies were confounded by limb unloading prior to resistance training, or (d) some studies used biochemical methods to assess myofibrillar protein concentrations that may yield inaccurate results. Moreover, with the exception of three studies (Cribb and Hayes, 2006; Cribb et al., 2007; Haun et al., 2019a), all of the aforementioned studies examined untrained participants, and all of these studies examined intramuscular protein responses to moderate-to-higher-volume training regimens. Thus, it is currently unknown how high-load resistance training affects skeletal muscle protein composition adaptations. The purpose of the present study was to examine intramuscular protein adaptations that occur in response to 10 weeks of high-load, low-volume, resistance training in previously-trained college-aged male participants. Specifically, fiber type-specific fCSA, myofibrillar protein concentrations, sarcoplasmic protein concentrations, myosin heavy chain and actin protein abundances, and muscle tissue percent fluid were assessed prior to and following the resistance training intervention. We recently used proteomics to report that 6 weeks of high-volume resistance training up-regulated creatine kinase as well as several glycolytic proteins (Haun et al., 2019a). Thus, as a secondary aim in the current study, we sought to examine the relative expression of individual sarcoplasmic proteins using shotgun proteomics analysis to determine if similar or different proteomic adaptations occurred with high- load training.

## Materials and Methods

### Ethics Approval and Participant Inclusion Criteria

All procedures in this study approved by the Institutional Review Board at Auburn University (Protocol #18-442 MR 1812), and this study conformed to the standards set by the latest revision of the Declaration of Helsinki.

Participants were verbally recruited at Auburn University. Eligible participants had to be free of cardiometabolic diseases (e.g., morbid obesity, type II diabetes, severe hypertension) or conditions precluding the collection of a skeletal muscle biopsy. Additionally, participants had to self-report resistance training for at least 12 months prior to the study. Interested participants provided verbal and written consent to participate prior to data collection procedures outlined below.

### Testing Sessions

**FIGURE 1:**
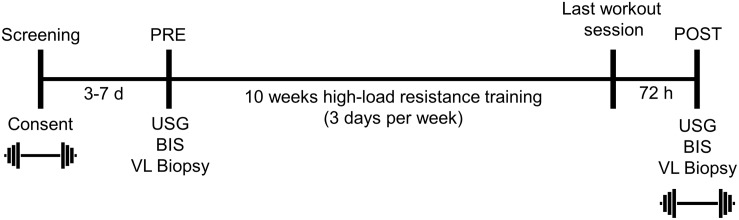
Study design. This figure illustrates the design and timing of data collection points for the study. USG, urine specific gravity; BIS, bioelectrical impedance spectroscopy; VL, vastus lateralis. Symbols: 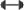, 3RM BB back squat testing.

The testing session detailed below occurred during the morning hours (0500–0900) following an overnight fast, and procedures are described in the order that they occurred. These sessions occurred prior to the 10-week training intervention (PRE), and 72 h following the last training bout (POST). A timeline of the study is provided in Figure 1 below.

#### Urine Specific Gravity Testing for Adequate Hydration

At the beginning of each testing session, participants submitted a urine sample (∼5 mL) for urine specific gravity (USG) assessment. A handheld refractometer (ATAGO; Bellevue, WA, United States) was used for measurements. USG measurements were performed given that being in a dehydrated state has been shown to affect body composition measures (Thompson et al., 1991), and being dehydrated likely affects muscle tissue fluid levels. Notably, all participants presented USG values ≤1.020 suggestive of a euhydrated state (American College of Sports et al., 2007) and, as a result, were considered adequately hydrated for further testing.

#### Bioelectrical Impedance Spectroscopy for Whole-Body Composition Assessment

Body composition was measured by bioelectrical impedance spectroscopy (BIS) using the SOZO device (ImpediMed Limited, QLD, Australia) according to the methods described by Moon et al. (2008). These methods have been previously shown by our laboratory to produce test-retest intraclass correlation coefficients (ICC) > 0.990 for whole body intracellular and extracellular water metrics on 24 participants (Haun et al., 2018), and this device estimates whole-body fat-free mass (FFM) based on these metrics.

#### Muscle Tissue Collection

Right leg vastus lateralis muscle biopsies were collected using a 5-gauge needle under local anesthesia as previously described (Haun et al., 2018; Roberts et al., 2018). Immediately following tissue procurement, ∼20–40 mg of tissue was embedded in cryomolds containing optimal cutting temperature (OCT) media (Tissue-Tek, Sakura Finetek Inc, Torrence, CA, United States). Embedding was performed according to methods previously reported from our laboratory where tissue was placed in cryomolds for cross-sectional slicing in a non-stretched state (Mobley et al., 2017a). Cryomolds were then frozen using liquid nitrogen-cooled isopentane and subsequently stored at −80°C until histology. The remaining tissue was teased of blood and connective tissue, wrapped in pre-labeled foils, flash frozen in liquid nitrogen, and subsequently stored at −80°C for other molecular analyses described below.

#### Three Repetition Maximum (3RM) Back Squat Strength Testing

Three repetition maximum back squat testing occurred 3–7 days prior to PRE testing and during POST testing following the skeletal muscle biopsy. Strict technical parameters were employed during back squat assessments under the direct supervision of the study coordinator who holds a Certified Strength and Conditioning Specialist Certification from the National Strength and Conditioning Association (NSCA). Testing first began with two rounds of calisthenics (jumping jacks, single-leg lunges). Participants then performed one warm-up set of 10 repetitions using the bar only (20 kg) followed by one set of 10 repetitions at ∼60 kg. Participants then performed three repetitions at a self-estimated 70% 1RM, and began maximal 3RM attempts thereafter. Approximately 3 min of rest was allowed between 3RM attempts, and repetitions were considered successful when a squat depth was accomplished where the hip crease was below the knee joint. 1RM values were estimated from 3RM values using the NSCA’s most recent text (Haff and Triplett, 2016).

### Training

Participants were provided a pre-programed high-load training regimen to adhere to over the 10-week intervention (Table 1). Participants began training by acclimating to the prescribed exercises over the course of 1 week. Thereafter, three identical blocks of training, lasting three weeks (weeks 2–10) each were prescribed. The prescribed training intensities for participants was based upon repetitions in reserve (RIR) from Helms et al. (2016), which has been shown to align well with a 1RM percentage. Participants were instructed to gauge lifting difficulty using the repetitions in reserve (RIR) scale immediately following each set of each exercise. The study coordinator explained that RIR was the number of repetitions the participant felt they could complete safely with proper technique beyond the number of repetitions assigned for the set. The target goal was for participants to rate each set at 2 RIR or less. If the participant perceived the load as being too light (e.g., >2 RIR) for a given set, they were instructed to increase the weight being utilized by ∼2.3–4.6 kg (5–10 lbs) for upper body movements, and ∼4.6–9.2 kg (10–20 lbs) for lower body movements. Conversely, if the participants perceived the load as being too heavy during a given set (e.g., 0 RIR or missed repetitions), participants were instructed to decrease weight by the aforementioned iterations on the subsequent set. Participants worked out at the Auburn University Student Recreation Center and were instructed to log training data daily using Google Sheets (Mountain View, CA, United States). The study coordinator verbally communicated with participants weekly to ensure that the training program was being conducted appropriately, and laboratory staff routinely spot-checked the recreational facility weekly to ensure participants were adherent to the training protocol.

**TABLE 1:** Prescribed training regimen.

Weeks	Day	Exercise	Sets × Reps	RIR (%1RM)
1	M	BB Squat, Pronated Grip Cable Pulldown	3 × 6	2(78%)
		DB Walking Lunge, Machine Hamstring Curl	3 × 8	2(73%)
		DB Reverse Fly, BB Curl	3 × 10	2(68%)
	T	BB Bench Press, Good Morning, Incline DB Bench Press	3 × 6	2(78%)
		Trap-bar Deadlift, DB Row	3 × 8	2(73%)
		Machine Triceps Extension	3 × 10	2(68%)
	R	BB Deadlift, BB Squat	3 × 6	4(72%)
		Supinated Grip Cable Pulldown	3 × 8	4(68%)
		Back Extension, DB Curl, DB Lateral Raise	3 × 10	4(66%)
	F	DB Bench Press, Supinated Grip BB Row	3 × 6	2(78%)
		Machine Hamstring Curl	3 × 8	2(73%)
		DB Skull Crusher, Reverse Fly	3 × 10	2(68%)
2, 5, 8	M	BB Squat, BB Deadlift, Machine Leg Extension	5 × 5	0(85%)
	W	BB Bench Press	3 × 4	2(83%)
		DB Rear Foot Elevated Split Squat	3 × 3	2(85%)
		Machine Hamstring Curl, DB Row, Back Extension	3 × 6	2(78%)
		DB Curl	3 × 8	2(73%)
	R	BB Squat	2 × 3	4(86%)
		Pronated Grip Pulldown, DB Skull Crusher	3 × 5	4(73%)
		DB Lateral Raise	3 × 6	4(72%)
		BB Shrug	3 × 8	4(68%)
	F	DB Bench Press	4 × 3	2(84%)
		Supinated Grip BB Row, Good Morning	4 × 5	2(80%)
		Back Extension	3 × 6	2(78%)
		BB Curl	3 × 8	2(73%)
3, 6, 9	M	BB Squat	3 × 4	2(83%)
		Pronated Grip Cable Pulldown, DB Walking Lunge	3 × 5	2(81%)
		Machine Hamstring Curl, DB Reverse Fly, BB Curl	3 × 6	2(78%)
	T	BB Bench Press	3 × 4	2(83%)
		Good Morning, Incline DB Bench Press, Trap-bar Deadlift, DB Row	3 × 5	2(81%)
		Machine Triceps Extension	3 × 6	2(78%)
	R	Deadlift, BB Squat	3 × 4	4(76%)
		Supinated Grip Pulldown, Back Extension	3 × 5	4(73%)
		DB Curl, Lateral Raise	3 × 6	4(72%)
	F	DB Bench Press, Supinated Grip BB Row	3 × 4	2(83%)
		Hamstring Curl, DB Skull Crusher	3 × 5	2(81%)
		Reverse Fly	3 × 6	2(78%)
4, 7, 10	M	BB Squat	4 × 2	1(90%)
		Pronated Grip Cable Pulldown, DB Walking Lunge	4 × 4	2(82%)
		Machine Hamstring Curl, DB Reverse Fly, BB Curl	4 × 5	2(80%)
	T	BB Bench Press	4 × 2	2(90%)
		Good Morning, Incline DB Bench Press, Trap-Bar Deadlift, DB Row	4 × 4	2(82%)
		Machine Triceps Extension	4 × 5	2(80%)
	R	BB Deadlift	4 × 2	1(90%)
		BB Squat	4 × 2	4(80%)
		Supinated Grip Cable Pulldown, Back Extension	4 × 4	4(75%)
		DB Curl, DB Lateral Raise	4 × 5	4(72%)
	F	DB Bench Press, Supinated Grip BB Row	4 × 2	2(87%)
		Machine Hamstring Curl, DB Skull Crusher	4 × 4	2(82%)
		Reverse Fly	4 × 5	2(80%)

*The table outlines the prescribed weekly training regimen for Monday (M), Tuesday (T), Wednesday (W), Thursday (R), and Friday (F). The last column is the prescribed intensity for participants based upon repetitions in reserve (RIR) from Helms et al. (2016); notably, the corresponding% one repetition maximum (%1 RM) is presented alongside these data in parentheses. BB, barbell; DB, dumbbell.*

### Biochemical Assays

#### Tissue Dehydration Prior to Biochemical Assays

Skeletal muscle foils were removed from −80°C storage, placed on a liquid nitrogen-cooled ceramic mortar and pestle, and tissue was pulverized into 2–4 mm^3^ chunks. Chunks (∼20 mg) were weighed using a scale with a sensitivity of 0.0001 g (Mettler-Toledo; Columbus, OH, United States) and placed into 1.7 mL polypropylene tubes with five ventilation holes drilled on the caps. Tissue in tubes were then freeze-dried using a laboratory-grade freeze-drying apparatus (FreeZone 2.5, Labconco; Kansas City, MO, United States). Freeze dry settings were −50°C for the condenser and a vacuum setting of 0.1 mBar, and samples were placed in the condenser well for 4 h under constant vacuum pressure. Following freeze drying, muscle was re-weighed on an analytical scale, and muscle fluid content was assessed using the following equation:

[(Prefreeze-driedmuscleweight-postfreeze-driedmuscleweight)/Prefreeze-driedmuscleweight]×100

This protocol was piloted using muscle tissue obtained from rat triceps (*n* = 5 independent tissue chucks) in one of our previous publications (Mobley et al., 2017b), and compared to overnight oven drying at 100°C (*n* = 5 independent tissue chucks). Both methods yielded similar tissue fluid content values (freeze-dry = 75.2%, oven = 74.5%; *p* = 0.355), and the freeze dry method produced a high degree of reliability (CV = 1.7%).

#### Sarcoplasmic and Myofibrillar Protein Isolation

Isolation of these protein fractions were performed similar to what has been previously described by our laboratory with slight modifications (Haun et al., 2019a). Immediately following post-lyophilization tissue weighing described above, dehydrated tissue (∼4–5 mg) was placed in new 1.7 mL tubes, and ice-cold buffer (300 μL; Buffer 1: 25 mM Tris, pH 7.2, 0.5% Triton X-100, protease inhibitors) was added to tubes. We ensured this process occurred rapidly in order to minimize tissue rehydration outside the lyophilizer. Samples were homogenized using tight-fitting pestles and centrifuged at 1,500 × *g* for 10 min at 4°C. Supernatants (sarcoplasmic fraction) were collected and placed in new 1.7 mL microtubes on ice. As a wash step, the resultant myofibrillar pellet was resuspended in 300 μL of Buffer 1 and centrifuged at 1,500 × *g* for 10 min at 4°C. The supernatant was discarded and the myofibrillar pellet was solubilized in 400 μL of ice-cold resuspension buffer (20 mM Tris–HCl, pH 7.2, 100 mM KCl, 20% glycerol, 1 mM DTT, 50 mM spermidine, protease inhibitors). The solubilized sarcoplasmic and myofibrillar fractions were then stored at −80°C until protein concentration determination and proteomic analyses described below. Notably, these methods differ slightly from what we have previously used, because of the difficulty in resuspending myofibrils. With extensive piloting, we determined the following: (a) using a different buffer 1 (in Haun et al., 2019a: 20 mM Tris–HCl, pH 7.2, 5 mM EGTA, 100 mM KCl, 1% Triton-X 100; in the current paper: 25 mM Tris, pH 7.2, 0.5% Triton X−100, protease inhibitors), led to less contamination of the sarcoplasmic fraction with actin and myosin, and (b) adding 50 mM spermidine to Buffer 2 herein (termed buffer 3 in Haun et al., 2019a) led to an increased solubilization of isolated myofibrils. While us and others have termed the first supernatant yielded from this method as the sarcoplasmic fraction (Moore et al., 2009; Brook et al., 2015; Haun et al., 2019a), it should noted that we have found this fraction to contain trace amounts of proteins that exist outside of muscle cells (e.g., albumin, hemoglobin from red blood cells) (Haun et al., 2019a).

#### Determination of Protein Concentration

Sarcoplasmic and myofibrillar protein resuspensions were batch-assayed for determination of protein concentration using a commercially-available bicinchoninic acid (BCA) kit (Thermo Fisher Scientific; Waltham, MA, United States). Samples were assayed in duplicate (sarcoplasmic protein) or triplicate (myofibrillar protein) using a microplate assay protocol where a small volume of sample was assayed (20 μL of 5x diluted sample + 200 μL Reagent A + B). The myofibrillar resuspensions were then assayed for myosin heavy chain and actin protein abundances using SDS-PAGE and Coomassie staining, and the sarcoplasmic fraction underwent proteomics analysis described below. Average replicate coefficients of variation for myofibrillar protein concentrations and sarcoplasmic protein concentrations were 3.3 and 3.9%, respectively.

#### SDS-PAGE and Coomassie Staining for Relative Myosin Heavy Chain and Actin Abundances

Determination of myosin heavy chain and actin protein abundances were performed as previously described by our laboratory (Roberts et al., 2018; Haun et al., 2019a) and others (Cohen et al., 2009). Briefly, SDS-PAGE sample preps were made using 10 μL resuspended myofibrils, 65 μL distilled water (diH_2_O), and 25 μL 4x Laemmli buffer. Samples (5 μL) were then loaded on pre-casted gradient (4–15%) SDS-polyacrylamide gels in duplicate (Bio-Rad Laboratories) and subjected to electrophoresis at 180 V for 40 min using pre-made 1× SDS-PAGE running buffer (VWR; Randor, PA, United States). Following electrophoresis, gels were rinsed in diH_2_O for 15 min and immersed in Coomassie stain (LabSafe GEL Blue; G-Biosciences; St. Louis, MO, United States) for 2 h. Gels were then destained in diH_2_O for 60 min, and bright field imaged using a handheld digital camera (iPhone 8S; Cupertino, CA, United States). Image files were converted to grayscale using a gel documentation system and associated software (UVP; Upland, CA, United States), and region of interest bands were drawn at the prominent 220 and 43 kD bands (representing myosin heavy chain and actin, respectively). Given that a standardized volume from all samples were loaded onto gels (5 μL), myosin heavy chain and actin protein band densities were normalized to input muscle weights to derive arbitrary density units (ADU) per mg dry muscle. All values were then divided by the mean of the PRE time point to depict myosin heavy chain and actin protein abundances. Contrary to conventional Western blotting methods where protein targets are normalized to total protein (e.g., Ponceau signal), this method allows researchers to determine the change in the relative abundances of myosin heavy chain and actin according to muscle weights. In scenarios where myosin heavy chain and actin protein band densities per muscle weight decrease in response to resistance training, this may be suggestive of a disproportional increase in muscle weight relative to myofibrillar protein accretion. Our laboratory has reported that this method yields exceptional sensitivity in detecting 5–25% increases in actin and myosin heavy chain protein abundances (Roberts et al., 2018). Average duplicate coefficients of variation for actin and myosin heavy chain protein abundances herein were 1.1 and 0.9%, respectively.

#### Proteomic Analysis of the Sarcoplasmic Fraction

Proteomics analysis was performed on the sarcoplasmic protein fraction similar to previous work published by our laboratory (Haun et al., 2019a). Each sample (90 μg) for triplicate technical runs (30 μg for each run) was prepared for LC-MS/MS analysis using EasyPep Mini MS Sample Prep Kit (Thermo Fisher Scientific). In brief, samples were transferred into new 1.7 mL tubes and final volumes were adjusted to 100 μL with general cell lysis buffer (Cell Signaling; Danvers, MA, United States). Reduction solution (50 μL) and alkylation solution (50 μL) were added to samples, gently mixed, and incubated at 95°C using a heat block for 10 min to reduce and alkylate samples. After incubation, the sample was removed from the heat block and cooled to room temperature. A reconstituted enzyme Trypsin/Lys-C Protease Mix solution (50 μL) was added to the reduced and alkylated protein sample and incubated with shaking at 37°C for 2 h to digest proteins. Digestion stop solution (50 μL) was then added to samples and peptides were cleaned using a peptide clean-up column according to the kit instructions. An externally calibrated Thermo Q Exactive HF (high-resolution electrospray tandem mass spectrometer) was used in conjunction with Dionex UltiMate3000 RSLC Nano System (Thermo Fisher Scientific). Samples (5 μL) were aspirated into a 50 μL loop and loaded onto the trap column (Thermo μ-Precolumn 5 mm, with nanoViper tubing 30 μm i.d. × 10 cm). The flow rate was set to 300 nL/min for separation on the analytical column (Acclaim pepmap RSLC 75 μM × 15 cm nanoviper; Thermo Fisher Scientific). Mobile phase A was composed of 99.9% H_2_O (EMD Omni Solvent; Millipore, Austin, TX, United States), and 0.1% formic acid and mobile phase B was composed of 99.9% acetonitrile and 0.1% formic acid. A 60-minute linear gradient from 3 to 45% B was performed. The eluent was directly nanosprayed into the mass-spectrometer. During chromatographic separation, the Q-Exactive HF was operated in a data-dependent mode and under direct control of the Thermo Excalibur 3.1.66 (Thermo Fisher Scientific). The MS data were acquired using the following parameters: 20 data-dependent collisional-induced-dissociation (CID) MS/MS scans per full scan (350 to 1700 m/z) at 60,000 resolution. MS2 were acquired in centroid mode at 15,000 resolution. Ions with a single charge or charges more than seven as well as unassigned charge were excluded. A 15-second dynamic exclusion window was used. All measurements were performed at room temperature, and three technical replicates were run for each sample. Raw files were analyzed using Proteome Discoverer (version 2.0, Thermo Fisher Scientific) software package with SequestHT and Mascot search nodes using the 20180308HumanSwissprot.fasta database and the Percolator peptide validator. The resulting.msf files were further analyzed by the proteome validator software Scaffold v4.0 (Portland, OR, United States). Final data for each protein were obtained as spectra data normalized to total spectra, and data are presented as such. Proteins at POST which deviated from PRE values (i.e., up- or down-regulated, *p* < 0.05) are presented in the results.

#### fCSA Analysis

Methods for immunohistochemistry were employed as previously reported by our laboratory and described elsewhere (Hyatt et al., 2015; Martin et al., 2016; Mobley et al., 2017a). Briefly, sections from OCT−preserved samples were cut at a thickness of 10 μm using a cryotome (Leica Biosystems; Buffalo Grove, IL, United States) and were adhered to positively-charged histology slides. Once all samples were sectioned, samples were batch-processed for immunohistochemistry. During batch processing sections were air-dried at room temperature for up to 10 min, and blocked with 100% Pierce Super Blocker (Thermo Fisher Scientific) for 25 min. Sections were then incubated for 60 min with a pre-diluted commercially-available rabbit anti-dystrophin IgG1 antibody solution (catalog #: GTX15277; Genetex Inc., Irvine, CA, United States) and spiked in mouse anti-myosin I IgG1 (catalog #: A4.951 supernatant; Hybridoma Bank, Iowa City, IA, United States; 40 μL added per 1 mL of dystrophin antibody solution, or a 1:25 dilution). Sections were then washed for 2 min in PBS and incubated in the dark for 60 min with a secondary antibody solution containing Texas Red-conjugated anti-rabbit IgG1 (catalog #: TI-1000; Vector Laboratories, Burlingame, CA, United States), and Alexa Fluor 488-conjugated anti-mouse IgG1 (catalog #: A-11001; Thermo Fisher Scientific) (∼6.6 μL of all secondary antibodies per 1 mL of blocking solution, or a 1:150 dilution). Sections were washed for 5 min in PBS, air-dried, and mounted with fluorescent media containing 4,6-diamidino-2-phenylindole (DAPI; catalog #: GTX16206; Genetex Inc.). Following mounting, digital images were immediately captured with a fluorescent microscope (Nikon Instruments, Melville, NY, United States) using a 10x objective. Approximate exposure times were 600 ms for TRITC and FITC imaging and 80 ms for DAPI imaging. This staining method allowed the identification of cell membranes (detected by the Texas Red filter), type I fiber green cell bodies (detected by the FITC filter), type II fiber black cell bodies (unlabeled), and myonuclei (detected by the DAPI filter). Standardized measurements of type I, type II, and mean fCSAs were performed using open-sourced software (MyoVision) (Wen et al., 2018). A pixel conversion ratio value of 0.493 was used to account for the size and bit-depth of images, and a detection range of detection from 200 to 12,000 μm^2^ was used to ensure artifact was removed (e.g., large fibers which may have not been in transverse orientation, or structures between dystrophin stains which were likely small vessels).

### Statistics

Statistical analyses were performed in SPSS (Version 25; IBM SPSS Statistics Software, Chicago, IL, United States) and RStudio (Version 1.1.463, R Foundation for Statistical Computing, Vienna, AT, UNited States). Normal distribution of dependent variables was performed using Shapiro-Wilks tests. Non-normally distributed data was log-transformed prior to statistical analysis. Dependent samples *t*-tests were performed when comparing dependent variables between the PRE and POST time points, and statistical significance was set at *p* < 0.05. BIS-derived extracellular water (ECW), and intracellular water (ICW) failed normality testing and were not correctable with log transformation, square root transformation nor cube root transformation. Thus, we performed both parametric and non-parametric comparisons on these variables and proceeded with parametric tests for all other dependent variables. Pearson correlations were also performed on select variables. For proteomics data, proteins that exhibited a significant up- or down-regulation from PRE to POST (unadjusted *p*-value < 0.05) were analyzed for functionally annotated pathways using the OFFICIAL_GENE_SYMBOL query in DAVID v6.8 (Huang et al., 2007). Notably, we chose to use unadjusted *p*-values for proteomics data given that: (a) Bonferroni-corrected *p*-values values would likely yield stringent adjusted *p*-values, and (b) Benjamini-Hochberg-adjusted *p*-values would likely yield *p*-values that were lower than un-adjusted *p*-values due to there being only 385 proteomic targets. All data herein are presented in figures and tables as means ± standard deviation values.

## Results

### Participant Baseline Characteristics and Training Adherence

The participants’ average age was 23 ± 3 years, self-reported training age was 6 ± 5 years (range: 1.5 to 16 years), pre-intervention fat-free mass index was 21.6 ± 2.7 kg/m^2^, and pre-intervention body fat percentage was 21.2 ± 4.7%. Of the 15 participants, nine individuals logged all of their workouts, four individuals logged >80% of their workouts, and two participants logged fewer than 80% of their workouts. For the two participants that poorly logged workouts, these individuals verbally confirmed that workouts were being completed, but cited time constraints for not completing training logs. Training data for the 13 participants that logged at least 80% of their workouts are presented in Table 2.

**TABLE 2:** Training volume data.

Week	*n*-size of those that logged	Lower Body Volume (kg)	Upper Body Volume (kg)
1	11	12,145 ± 2,553	15,830 ± 3,951
2	13	10,807 ± 3,729	10,917 ± 4,152
3	13	9,013 ± 2,969	11,027 ± 3,706
4	13	8,190 ± 2,864	10,697 ± 4,251
5	13	10,421 ± 3,538	12,898 ± 3,723
6	13	9,268 ± 3,722	10,786 ± 4,367
7	11	8,783 ± 3,551	11,928 ± 3,162
8	13	10,187 ± 3,513	12,578 ± 3,465
9	12	9,516 ± 2,669	10,793 ± 4,345
10	9	9,505 ± 2,935	12,181 ± 3,852

*These data are from participants who continuously logged workouts throughout the study. Notably, all 15 participants verbally confirmed completing the prescribed workouts, 13 participants logged >80% of all training data, and 9 participants logged all training sessions.*

### Body Composition and 1RM Squat Strength Adaptations

PRE- and POST-intervention body composition and estimated 1RM squat strength for all participants are presented in Table 3. A decrease in fat-free mass (*p* = 0.026), and an increase in squat strength (*p* < 0.001) were observed. However, parametric statistics indicated no significant changes occurred in ECW (PRE: 23.0 ± 3.7 L, POST: 22.4 ± 3.2 L, *p* = 0.087) or ICW (PRE: 29.0 ± 4.6 L, POST: 28.8 ± 4.3 L, *p* = 0.365) (data not shown). Given these variables were the only variables that non-normally distributed, we also performed Wilcoxon signed-rank tests. These tests also indicated no significant changes occurred in ECW (*p* = 0.131) or ICW (0.410).

**TABLE 3:** Pre- and post-intervention body composition and 3RM squat strength.

Variable	PRE (mean ± SD)	POST (mean ± SD)	*p*-value
FFM (kg)	71.0 ± 11.3	69.9 ± 10.2	0.026
Fat mass (kg)	19.5 ± 6.2	19.5 ± 6.4	1.00
Est. 1RM squat (kg)	140 ± 27	159 ± 30	<0.001

*FFM, fat-free mass; Est. 1RM, 1RM back squat estimated from 3RM testing.*

### fCSA Adaptations

**FIGURE 2:**
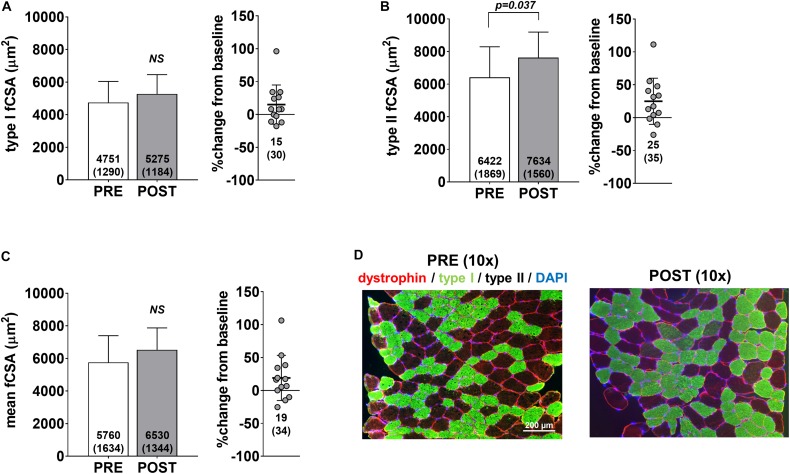
fCSA adaptations to high-load training. PRE to POST values for type I fCSA **(A)**, type II fCSA **(B)**, and mean fCSA **(C)**. Panel **D** contains 10× representative PRE and POST images from a participant (scale bar = 200 μm).

Pre- and post-intervention type I, type II and mean fCSAs are presented in Figure 2. Notably, only 13 of the 15 participants yielded tissue of adequate quality to perform immunohistochemical analysis. No significant changes in type I fCSA (*p* = 0.104) or mean fCSA were observed (*p* = 0.110) (Figures 2A,C, respectively), although an increase in type II fCSA was observed (*p* = 0.028) (Figure 2B). Minimum feret diameters for type I fibers were 63.7 ± 9.0 μm at PRE and 68.3 ± 8.5 μm at POST (*p* = 0.085), whereas values for type II fibers were 74.1 ± 10.5 μm at PRE and 81.9 ± 8.3 μm at POST (*p* = 0.020) (data not shown). Fiber counts averaged to be 105 ± 32 at PRE and 108 ± 27 at POST.

### Skeletal Muscle Protein Adaptations

**FIGURE 3:**
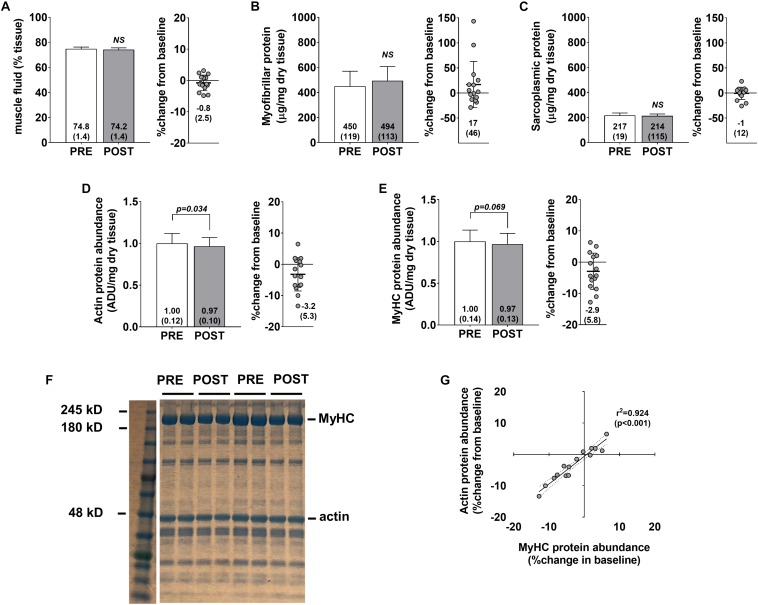
Skeletal muscle protein composition adaptations to high-load training. PRE and POST values for muscle percent fluid **(A)**, myofibrillar protein concentrations **(B)**, sarcoplasmic protein concentrations **(C)**, actin protein abundance **(D)**, and myosin heavy chain protein abundance **(E)**. **(F)** Demonstrates representative Coomassie PRE and POST images from two participants. **(G)** Demonstrates the association between actin and myosin heavy chain protein abundance changes from PRE to POST (regression lines are solid and 95% confidence interval bands are dashed).

No significant changes were observed for muscle percent fluid (*p* = 0.236) (Figure 3A), myofibrillar protein concentrations (*p* = 0.258) (Figure 3B), or sarcoplasmic protein concentrations (*p* = 0.654) (Figure 3C). Interestingly, a modest but significant decrease in actin protein abundance was observed (*p* = 0.034) (Figure 3D), and a decrease in myosin heavy chain protein abundance approached statistical significance (*p* = 0.069) (Figure 3E). Pre- to post-intervention percent changes in actin and myosin heavy chain protein abundances were highly correlated (*r*^2^ = 0.924, *p* < 0.001) (Figure 3G) suggesting that the coupling of these proteins is highly conserved. However, positive non-significant associations were observed for percent change in myofibrillar protein concentrations versus percent change in myosin heavy chain (*r*^2^ = 0.209, *p* = 0.086) and percent change in actin (*r*^2^ = 0.262, *p* = 0.051) (data not shown). Indeed, changes in myofibrillar proteins other than myosin heavy chain and actin may explain these poor associations. However, we believe that these relationships are likely due to the poorer sensitivity of the BCA assay in quantifying myofibrillar proteins and this is discussed in detail below.

### Associations Between Pre-training Protein Concentrations and Percent Changes in These Metrics

**FIGURE 4:**
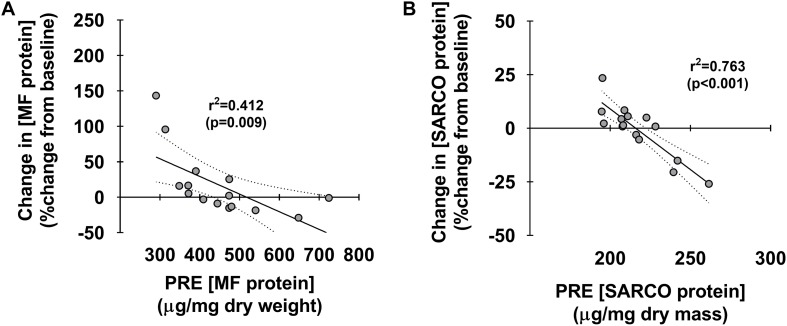
Associations between pre-training muscle protein levels versus percent change in these metrics. Associations between pre-training myofibrillar and sarcoplasmic protein concentrations versus PRE to POST percent changes in these metrics (**A,B**, respectively). For each panel, regression lines are solid and 95% confidence interval bands are dashed.

Figure 4 depicts associations between pre-training myofibrillar and sarcoplasmic protein concentrations and percent changes in these metrics. Interestingly, both pre-training myofibrillar (*r*^2^ = 0.412, *p* < 0.009) and sarcoplasmic protein concentrations (*r*^2^ = 0.763, *p* < 0.001) were both negatively associated with the PRE-to-POST percent change in these metrics (Figures 4A,B, respectively).

### Individual Sarcoplasmic Protein Adaptations

Our bioinformatics enabled us to examine 536 annotated protein targets that displayed a spectra signal in one of the 10 participants at PRE and/or POST. This list was refined to 385 targets after grouping some of the annotated targets into clusters (e.g., “Cluster of beta-enolase” contained composite spectra values of ENO1/2/3). Of this list of 385 targets, the top enriched proteins or protein clusters at PRE that constituted >1.0% of the total spectra are depicted in Table 4. This list confirmed that most of the proteins were intracellular proteins, although there was a presence of hemoglobin-alpha and -beta subunits along with serum albumin, which was likely due to residual blood being in the tissue prior to processing. Additionally, 87 of the 385 targets were expressed in all participants at PRE, whereas 31 of the 385 targets were not expressed in any of the participants at PRE and were lowly expressed in certain participants at POST.

**TABLE 4:** Top enriched proteins in the sarcoplasmic fraction prior to training.

Protein (acronym)	MW	% of total protein pool
Creatine kinase M-type (CKM)	43 kDa	11.1
Myoglobin (MB)	17 kDa	8.2
Cluster of Glyceraldehyde-3-phosphate dehydrogenase (GAPDH)	36 kDa	7.1
Cluster of Hemoglobin subunit beta (HBB)^∗^	16 kDa	6.4
Cluster of Hemoglobin subunit alpha (HBA1)^∗^	15 kDa	4.8
Cluster of Beta-enolase (ENO3)	47 kDa	4.5
Serum albumin (ALB)^∗^	69 kDa	4.4
Fructose-bisphosphate aldolase A (ALDOA)	39 kDa	4.3
Cluster of Glycogen phosphorylase (PYGM)	97 kDa	3.6
Carbonic anhydrase 3 (CA3)	30 kDa	3.0
Cluster of Pyruvate kinase (PKM)	58 kDa	2.7
Triosephosphate isomerase (TPI1)	31 kDa	2.0
Cluster of Phosphoglycerate kinase 1 (PGK1)	45 kDa	1.9
Cluster of Actin, alpha (ACTA1)	42 kDa	1.8
Sarcoplasmic/endoplasmic reticulum calcium ATPase 1 (ATP2A1)	110 kDa	1.7
Cluster of Phosphoglycerate mutase 2 (PGAM2)	29 kDa	1.6
Cluster of L-lactate dehydrogenase A chain (LDHA)	37 kDa	1.5
Phosphoglucomutase-1 (PGM1)	61 kDa	1.2
Adenylate kinase isoenzyme 1 (AK1)	22 kDa	1.1
ATP synthase subunit beta, mitochondrial (ATP5F1B)	57 kDa	1.1
Heat shock protein beta-1 (HSPB1)	23 kDa	1.0
Sarcoplasmic/endoplasmic reticulum calcium ATPase 2 (ATP2A2)	115 kDa	1.0
ATP synthase subunit alpha, mitochondrial (ATP5F1A)	60 kDa	1.0
Alpha-crystallin B chain (CRYAB)	20 kDa	1.0

*These data display the top enriched proteins in the sarcoplasmic fraction at PRE of the 10 participants who were interrogated using proteomics (>1.0% of total spectra). Notably, 21 of the 24 targets are intracellular proteins, whereas three of the targets (HBB, HBA1, ALB) are serum/blood proteins (indicated by “^∗^”). The presence of these three proteins indicates marginal contamination from blood embedded in tissue samples prior to tissue processing.*

**FIGURE 5:**
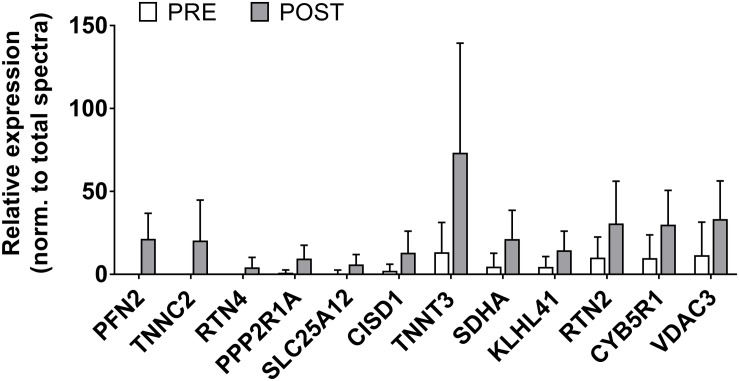
Sarcoplasmic protein adaptations to high-load training. This figure illustrates that the relative abundance of 12 sarcoplasmic proteins increased (*p* < 0.05) with high-load training. These proteins include profilin-2 (PFN2), troponin C (TNNC2), reticulon-4 (RTN4), serine/threonine protein phosphatase 2A (PPP2R1A), calcium-binding mitochondrial carrier protein Aralar1 (SLC25A12), CDGSH iron-sulfur domain-containing protein 1 (CISD1), troponin T (TNNT3), succinate dehydrogenase [ubiquinone] flavoprotein subunit, mitochondrial (SDHA), Kelch-like protein 41 (KLHL41), reticulon-2 (RTN2), NADH-cytochrome b5 reductase 1 (CYB5R1), and voltage-dependent anion-selective channel protein 3 (VDAC3).

There were 12 sarcoplasmic proteins that were up-regulated from PRE to POST (unadjusted *p* < 0.05 from PRE to POST; Figure 5). We queried functional pathways for the 12 up-regulated proteins using DAVID v6.8, but no significant pathways were unveiled. One protein was down-regulated from PRE to POST (HBA1, or hemoglobin subunit alpha; relative expression normalized to total spectra at PRE = 2382 ± 1021 AU and POST = 1378 ± 628 AU, *p* = 0.018). All proteomics data can be found in Supplementary File S1.

## Discussion

The purpose of this study was to examine skeletal muscle protein composition adaptations that occur in response to 10 weeks of high-load, moderate-volume resistance training in previously-trained college-aged male participants. Our data suggest 10 weeks of high-load resistance training facilitates the following adaptations in these participants: (a) a robust increase in lower body strength, (b) a modest, but significant decrease in whole body FFM, (c) a significant increase in type II, but not type I, fCSA, (d) a modest, but significant, decrease in skeletal muscle actin protein abundance and a similar statistical trend with myosin heavy chain protein abundance, (e) no changes in muscle fluid content or sarcoplasmic protein concentrations, and (f) marginal alterations in the sarcoplasmic proteome, with only 13 proteins being altered from PRE to POST (12 up, 1 down, unadjusted *p* < 0.05).

The observed increases in type II fCSA with high-load resistance training agree with numerous studies. For instance, a 2004 review article by Fry (2004) suggests high-load training protocols (e.g., protocols that implement 85% 1RM loads) preferentially increase type II fCSA relative to lower-load training (e.g., protocols that implement 60% 1RM loads). Several previous reports also suggested that type II muscle fibers preferentially hypertrophy in response to high-load training over an 8-week period (Campos et al., 2002; Schuenke et al., 2012; Netreba et al., 2013). While insightful, none of these aforementioned studies comprehensively examined skeletal muscle protein composition adaptations that occurred in response to high-load training. In this regard, one of the more provocative findings herein is the observed decrements in actin and myosin heavy chain protein abundances from after training. From a conceptual standpoint, no change in relative actin or myosin heavy chain abundances during training-induced skeletal muscle hypertrophy indicates that tissue or cell size increases coincided with a proportional accretion of contractile protein. On the other hand, increases or decreases in relative actin or myosin heavy chain abundances with muscle hypertrophy would potentially indicate the occurrence of myofibrillar packing (i.e., a higher density of myofibrils per fiber in cross section) or disproportionate sarcoplasmic expansion (i.e., a higher density of myofibrils per fiber in cross section), respectively.

Our laboratory has speculated that a decrease in relative actin or myosin heavy chain abundances during training-induced skeletal muscle hypertrophy as being indicative of “sarcoplasmic hypertrophy.” While sarcoplasmic hypertrophy research is in its infancy and is not a well-established phenomenon, it is notable that this is not the first investigation to suggest resistance training decreases skeletal muscle actin and myosin and myosin heavy chain protein abundances. Notably, our team recently reported that 6 weeks of high volume resistance training significantly reduces actin and myosin heavy chain protein abundances by ∼30%, while increasing mean fCSA by 23% in previously-trained college-aged males (Haun et al., 2019a). Others have also used transmission electron microscopy to demonstrate resistance training increases fCSA through cytoplasmic expansion (Macdougall et al., 1982; Toth et al., 2012). These collective findings have led us to speculate high-volume resistance training may facilitate fCSA increases predominantly through sarcoplasmic hypertrophy, and that this phenomenon “bioenergetically” and spatially primes cells for eventual myofibrillar protein accretion. However, our interpretations of the Haun et al. (2019a) data are limited in that the training volume utilized in that study was extraordinarily high, and it is unlikely that such a training paradigm would be utilized in real-world scenarios. Furthermore, while actin and myosin heavy chain protein abundances decreased in the current study, PRE-to-POST changes were only ∼3% for both proteins. Concentration decrements in both contractile proteins were not nearly as robust as observed in Haun et al. (2019a) study and, in lieu of the 19% increases in type II fCSA herein, these data suggest appreciable myofibrillar protein accretion likely occurred with the implemented high-load training program. It is notable that data from two studies by Cribb et al. (2007) contradict the aforementioned paradigm. In the first study the authors reported that 10 weeks of resistance training with or without the timing of nutrient provision surrounding workouts increased contractile protein content from ∼65 mg/g wet muscle to ∼90 mg/g wet muscle (Cribb and Hayes, 2006). In the second study participants were examined prior to and following 10 weeks of resistance training while using different nutritional supplements (Cribb et al., 2007). Only change scores in myofibrillar protein content were reported, and similar to the first publication, the authors noted that PRE-to-POST intervention changes across groups were ∼20–30 mg/g wet muscle. Both findings suggest that a large degree of myofibril packing occurred with training. However, the methods used to determine contractile protein content were not specified in either publication, and we (Vann et al., 2020) and others (Haus et al., 2007) have data suggesting myofibrillar protein concentrations are almost two-fold higher in younger untrained individuals (∼100–130 mg/g wet muscle) when compared to their participants’ baseline values. Given these disparities in findings between laboratories, more research is warranted in determining how resistance training affects contractile protein concentrations in skeletal muscle.

We elected to perform shotgun proteomics analysis on the sarcoplasmic fraction of muscle tissue, rather than the myofibrillar fraction, given that the relative abundances of the two predominant myofibrillar proteins (i.e., actin and myosin) were assessed using SDS-PAGE and Coomassie staining. Further, while we have previously examined how 6 weeks of high-volume resistance training affected the sarcoplasmic proteome (Haun et al., 2019a), the alteration in individual sarcoplasmic proteins in response to high-load training remain unknown. Contrary to our previous report where 40 sarcoplasmic proteins were significantly up-regulated (unadjusted *p* < 0.05), in the current study only 12 sarcoplasmic proteins were up-regulated following 10 weeks of high-load training. As stated prior, we queried functional pathways for the 12 up-regulated proteins, but no significant pathways were unveiled. Instead of putting forth speculations of what the change in each of the individual 12 proteins could potentially signify, we elected to discuss the broader theme of our proteomics analysis data relative to the findings from Haun et al. (2019a). When comparing these two studies, key differences in training methodology included having participants engage in 6 versus 10 weeks of training, lower body training volume being appreciably greater in the previous report than the current study, and training load being greater in the current study than the previous report which implemented 60% 1RM lifts. To this point, the prescribed load for all exercises on the last week of training in the Haun et al. (2019a) study averaged to be 86,694 ± 13,397 kg, whereas the prescribed load for all exercises on the last week of training in the current study was approximately 4-fold lower (21,686 ± 6,654 kg). Additionally, the post-study biopsy in the Haun et al. (2019a) paper was 24 h after the last high volume training bout, whereas the POST biopsy here occurred 72 h following the last training bout. As indicated in Table 5 below, the higher volume training protocol implemented in the previous report increased the expression of numerous sarcoplasmic and mitochondrial proteins related to glycolysis and ATP generation. While a few mitochondrial-related proteins were up-regulated with high-load training (e.g., SDHA, SLC25A12), none of the proteins presented in Table 5 were affected in the current study. Further, bioinformatics indicated that the glycolytic pathway was significantly upregulated with high volume training protocol in the previous study, whereas no functional pathways were affected with high-load training. These two proteomic interrogations from our laboratory provide potential evidence to suggest: (a) adaptations in the ATP-PCr and glycolytic energy systems occur in skeletal muscle in response to high-volume resistance training, and (b) high-load resistance training, while causing increases in strength and type II fiber hypertrophy, promotes a different proteomic adaptation than what was observed with high volume training. We posit that these proteomic signatures suggest greater metabolic adaptation (in the form of increased glycolysis) may occur with high volume training. This hypothesis is not far-reaching given the recent data from Lim et al. (2019) who reported 10 weeks of high volume resistance training increases mitochondrial adaptations to a greater degree compared to high-load training. However, given the design and analytical differences between our studies, we are limited in this interpretation. Therefore, more research is needed in order to determine if differences in volume-load during resistance training plays an appreciable role in facilitating metabolic adaptation.

**TABLE 5:** Changes in sarcoplasmic metabolic proteins that we previously reported to be significantly up-regulated in response to 6 weeks of high-volume resistance training.

Protein	PRE	POST	*p*-value
***ATP-PCr and adenylate kinase pathways***			
Creatine kinase M-type	5567 ± 1347	5784 ± 1364	0.743
Adenylate kinase	563 ± 117	591 ± 111	0.637
***Glycolysis***			
Beta-enolase	2224 ± 635	2249 ± 749	0.508
Fructose-bisphosphate aldolase A	2136 ± 659	2107 ± 650	0.930
Triosephosphate isomerase	983 ± 267	967 ± 211	0.873
Glyceraldehyde-3-phosphate dehydrogenase	3570 ± 992	3689 ± 1081	0.809
Fructose-bisphosphate aldolase C	64 ± 65	97 ± 67	0.328
Phosphoglycerate kinase 1	958 ± 291	1063 ± 266	0.466
L-lactate dehydrogenase A	744 ± 242	712 ± 289	0.722
ATP-dependent 6-phosphofructokinase	292 ± 155	426 ± 231	0.205
***Mitochondrial ATP generation***			
ATP synthase subunit beta	559 ± 220	658 ± 287	0.456
ATP synthase subunit alpha	486 ± 220	592 ± 287	0.435
Cytochrome c	21 ± 17	31 ± 20	0.272
ADP/ATP translocase	159 ± 75	188 ± 89	0.517

*This list was derived from previous data from our laboratory (Haun et al., 2019a) which demonstrated that 6 weeks of high-volume resistance training significantly upregulated muscle sarcoplasmic proteins involved with ATP generation. Notably, the high-load training utilized herein did not affect the expression of these proteins. All data represent expression values relative to total spectra (mean ± SD) from LC-MS/MS analysis.*

Finally, data in Figure 4 demonstrate that individuals who began training with lower myofibrillar and sarcoplasmic protein concentrations generally presented more robust increases in these metrics with 10 weeks of high-load resistance training. This is the third occurrence where we have observed this phenomenon (Roberts et al., 2018; Haun et al., 2019a), and suggests that individuals with low pre-training skeletal muscle protein concentrations may experience more robust increases in sarcoplasmic or myofibrillar protein accretion in response to training. Alternatively stated, our data imply that individuals who begin training with higher skeletal muscle protein concentrations may experience lower increases in synthesis rates or accretion. This notion of a “proteinstat” mechanism regulating muscle cell growth is not novel. In this regard, Millward authored an extensive review positing skeletal muscle possesses an extracellular proteinstat mechanism which limits intracellular protein accretion (Millward, 1995). The author put forth a “Bag-theory” of intracellular protein accretion in which muscle cells behave like bags and, when filled with protein to capacity, can only continue to grow if the bag (i.e., extracellular matrix) undergoes a remodeling process. Others have since theorized protein accretion in mitotic cells reach a size threshold prior to triggering cellular division (Soltani et al., 2016), which further supports the notion of a conserved proteinstat mechanism across a variety of cells. While this hypothesis is compelling for skeletal muscle, the data needed to support such a theory are lacking and tracer studies are needed in order to determine if pre-training intracellular protein concentrations are inversely related to the fractional synthetic response to resistance training.

### Limitations and Experimental Considerations

While this study provides novel data regarding skeletal muscle protein composition adaptations to high-load resistance training, there are limitations. First, although training was prescribed and monitored, direct supervision was not implemented. Notwithstanding, we do have good reason to believe participants were compliant due to weekly communication as well as online logging as described above. Additionally, only one training intervention was utilized. In this regard, future research is needed to compare the protein markers assessed herein with high-load versus high-volume training within or between subjects. The training paradigm itself was relatively complex in both exercise selection as well as prescribed load. The intent of this paradigm was to maintain the participants’ interest in adhering to the study while increasing training load. Although both were accomplished, our training paradigm is more difficult to reproduce given the complexity and this is a limitation. There was also a high degree of variation in self-reported training age (range: 1.5–16 years), so this too is another limitation. There were slight differences in buffers used to isolate sarcoplasmic proteins between the current study and Haun et al. (2019a), and these differences are discussed in the methods. While we posit that the removal of EGTA and KCl did not have an appreciable influence on the isolation of sarcoplasmic proteins, this is a possibility and unresolved limitation. Finally, the implemented mass spectrometry parameters only allowed for the identification of 536 sarcoplasmic proteins (385 after clustering). It should be noted that, while this does allow for the identification of highly-abundant proteins in the lysates, the relative coverage is low compared to other laboratories who implemented different sample processing and analytical techniques (Murgia et al., 2015; Schiaffino et al., 2019). Thus, it remains possible that high load training affected the expression of numerous low abundant proteins that were not detected herein.

One notable finding is the disagreement between our BCA assay and Coomassie data. Notably, the Coomassie data suggests high-load training slightly reduced the relative abundances of actin and myosin (per mg dry tissue), whereas the BCA data suggest that no statstical change in myofibrillar protein concentrations (per mg dry tissue) occurred. While this finding is difficult to reconcile, we feel the BCA assay has limitations. First, the principle of the BCA assay relies upon the presence of only four amino acids in the sample (cysteine, tyrosine, and tryptophan side chains) (Walker, 1994). Conversely, Coomassie reagent interacts with all positive amine groups in the sample (Brunelle and Green, 2014), and should account for all amino acids. Additionally, our data herein show that the CV values with the Coomassie method demonstrate a higher degree of sensitivity compared to the CV values yielded by the BCA assay and, as mentioned previously, our Coomassie method provides good sensitivity for detecting small changes in myosin heavy chain and actin (Roberts et al., 2018). Finally, it is notable that the PRE-to-POST percent change in myofibrillar protein concentrations were larger and more variable (+ 17 ± 46%) compared to percent change in myosin heavy chain and actin concentrations (∼−3 ± 5%). While our laboratory and others have used the BCA assay in detecting changes in myofibrillar protein concentrations during exercise training studies (Carrithers et al., 2002; Harber et al., 2009; Lemoine et al., 2009; Reidy et al., 2017; Roberts et al., 2018), these findings question the validity and sensitivity of using the BCA assay for quantifying myofibrillar protein changes. Instead, we propose researchers should consider using SDS-PAGE and Coomassie staining for detecting changes in myosin heavy chain and actin band densities given the aforementioned arguments put forth. It is also notable that we observed a significant decrease in FFM following the training intervention as determined by BIS. While small (∼ 1 kg) we posit that this was likely due to fluid shifts that occurred given that the BIS uses whole-body ICW and ECW to extrapolate body composition. Alternatively stated, we do not believe that participants experienced muscle atrophy. In support of this hypothesis, our histology data indicated type II fiber hypertrophy preferentially occurred herein. Likewise, ECW values trended downward from PRE to POST (*p* = 0.086) and this could have explained why FFM values were lower at POST. Interestingly, the ECW changes observed herein may be related to implementation of low-volume lifting over the 10-week period. In this regard, we recently reported that the high volume lifting implemented in our previous study by Haun et al. (2019a) led to appreciable increases in whole-body ECW (Haun et al., 2018). Thus, these observations warrant future research examining how different volume-loads affect fluid shifts, and whether fluid shifts confound hypertrophy outcomes.

## Conclusion

This is one of only a handful of studies to comprehensively examine skeletal muscle protein composition adaptations that occur in response to resistance training in previously-trained individuals, and is the first study to examine how high-load training affects these variables. Given that data in this area are limited, more studies that examine how different loads affect the markers assayed herein are essential.

## Data Availability Statement

The proteomic data generated in this study have been deposited into the Proteome XChange database (accession: PXD017805).

## Ethics Statement

The studies involving human participants were reviewed and approved by Auburn University Institutional Review Board. The patients/participants provided their written informed consent to participate in this study.

## Author Contributions

CV and MR designed the study and primarily drafted the manuscript, and CF, PR, VB, and DB provided the critical edits. CV designed the training program for participants, coordinated the study, and performed all analyses except proteomics. VB, BM, and VI prepared the samples for proteomics. RS performed the proteomics analysis. All other authors either assisted with testing, assays, or other aspects of the study.

## Conflict of Interest

The authors declare that the research was conducted in the absence of any commercial or financial relationships that could be construed as a potential conflict of interest.
